# Bird cestodes from Huinay (Comau Fjord), Chilean Patagonia: several species of the family Dilepididae (Platyhelminthes, Cyclophyllidea), with the erection of two new genera

**DOI:** 10.3897/zookeys.797.28005

**Published:** 2018-11-19

**Authors:** Jean Mariaux, Boyko B. Georgiev

**Affiliations:** 1 Natural History Museum of Geneva, CP 6434, 1211 Geneva 6, Switzerland Natural History Museum of Geneva Geneva Switzerland; 2 Department of Genetics and Evolution, University of Geneva, Boulevard d’Ivoy 4, 1205 Geneva, Switzerland University of Geneva Geneva Switzerland; 3 Institute of Biodiversity and Ecosystem Research, Bulgarian Academy of Sciences, 2 Gagarin Street, 1113 Sofia, Bulgaria Institute of Biodiversity and Ecosystem Research, Bulgarian Academy of Sciences Sofia Bulgaria

**Keywords:** Biodiversity, Cestoda, Chile, COI, Dilepididae, Furnariidae, helminth parasites, Passeriformes, Rhinocryptidae, Tyrannidae

## Abstract

Birds in the Huinay area, Los Lagos region, Chile, were studied for parasites. Here we report 2 new genera and species of the family Dilepididae (Cyclophyllidea) found in common local passerines: *Janinelliapeebeehi***gen. n.**, **sp. n.** was found in *Elaeniaalbiceps* (Tyrannidae) and *Huinaylepiselegans***gen. n.**, **sp. n.** was found in *Aphrasturaspinicauda* (Furnariidae). Other dilepidid parasites are reported for the first time from *Xolmispyrope* (Tyrannidae) and from 2 species of Rhinocryptidae. *Cotylorhipissureshi* Jadhav & Shinde, 1981 is considered a species inquirenda. The very high diversity and endemism of the observed cestode fauna in the Valdivian temperate rain forests is noted.

## Introduction

Chile is home to a very rich avifauna comprising more than 460 species (Jaramillo et al. 2003). However, apart from a few isolated reports limited to a small number of hosts (i.e. Babero et al. 1981; Torres et al. 1991; González-Acuña et al. 2017), very little is known of the cestode fauna of birds in this country. In the frame of a global and coordinated effort to improve our knowledge of the diversity of the Cestoda (Platyhelminthes) (Caira and Jensen 2017), the authors spent two weeks collecting bird tapeworms in coastal Valdivian temperate rain forests around the Huinay Scientific Field Station (HSFS) (Chile) in November and December 2008. A total of 87 birds belonging to 19 species were examined for parasites during this trip and 31 of them (36%) belonging to 12 species (63%) were found infected with one or more species of cestode. The diversity and novelty of this parasitic fauna is surprisingly high: all the parasite taxa recovered are new for Chile and several are new for science. Two earlier contributions based on this material described a new hymenolepidid genus from Trochilidae (Widmer et al. 2013) as well as a new species of the genus *Anonchotaenia* (Paruterinidae) from the tyrant flycatcher *Elaeniaalbiceps* (Tyrannidae) (Phillips et al. 2014).

This paper reports on a series of tapeworms of the family Dilepididae found in hosts belonging to the Furnariidae, Tyrannidae and Rhinocryptidae, including 2 new genera and species.

## Methods

Hosts were caught with mist nets, kept in fabric bags for a short time, then killed with an overdose of diethyl-ether, and autopsied immediately after their death. The digestive system was entirely removed, cut in sections if necessary, and searched for parasites under a dissecting microscope. Cestodes were fixed in hot 4% formalin after a small fragment was preserved in ethanol for DNA studies. Fixed specimens were transferred to 70% ethanol for storage. Further treatment included staining with Mayer’s hydrochloric carmine, dehydrating in an ethanol series, clearing in eugenol and mounting in Canada balsam. Some scoleces were prepared in Berlese’s medium for examination of their rostellar armament.

Drawings and photographs were made respectively with a drawing tube and a digital camera on a Nikon 80i microscope. Unless otherwise stated all measurements are in micrometers. Minimum and maximum values are reported followed by the mean and number of observations in parentheses when applicable. Conventions for dilepidid descriptions follow Bona (1994). All material is deposited in the Invertebrates Department of the Museum of Natural History of Geneva (MHNG). The nomenclature of birds follows the latest available edition of Avibase (Lepage 2018). Partial COI (cox1) sequences were generated according to the methodology described in Scholz et al. (2013). These sequences are deposited in Genbank under accession numbers MH663460 to MH663465 (data by A. Waeschenbach, Natural History Museum London, UK).

Collections acronyms: MHNG-PLAT: Muséum d’Histoire Naturelle de Genève (Invertebrates Department, Platyhelminthes Collection), USNM: United States National Museum (Cestode Collection).

## Results

### Janinellia gen. n.

Taxon classificationAnimaliaCyclophyllideaDilepididae

http://zoobank.org/0E3A0865-7B2B-4A17-AF2A-ADF1F9D880B8

#### Type species.

*Janinelliapeebeehi* sp. n. by original designation.

#### Diagnosis.

Dilepididae, Dilepidinae. Body small to medium. Scolex without rostellum, apical structures consist of unarmed glandular pouch. Suckers weakly muscular. Genital pores irregularly alternating. Genital ducts passing between osmoregulatory canals. Cirrus sac weakly muscular, elongate. Cirrus unarmed. Vagina posterior to cirrus sac. Testes numerous, posterior, in one field. Uterus initially reticular. Parasite of South-American passerines (Tyrannidae).

#### Etymology.

The genus name (feminine) is dedicated to Prof. Janine N. Caira (Storrs, Connecticut, USA) in recognition of her remarkable and tireless action in favour of tapeworm systematics.

#### Remarks.

Dilepidids with distinct but unarmed apical structures are known from various avian hosts, and are presently classified within several genera as synthesized by Bona (1994). Their classification is difficult, as they are mostly known only from a few species (or even specimens) and show few distinctive characters. According to Bona (1994), they are presently attributed to genera according to their apical glandular or muscular structures. Most of them can easily be differentiated from the material described above: *Cotylorhipis* Blanchard, 1909 because of its armed suckers; *Unciunia* Skrjabin, 1914 because of its cirrus armament with a tuft of setae resembling those in the genus *Spiniglans* Yamaguti, 1959 (see Mariaux and Georgiev 2018 for a recent discussion); *Ptilotolepis* Spasskii, 1969 because its genital ducts pass the osmoregulatory canals dorsally and egg capsules; *Platyscolex* Spasskaya, 1962 because its genital ducts are dorsal to the osmoregulatory canals, scolex shape, genital atrium structure and testis distribution; *Eburneotaenia* Bona, 1994 (see Mariaux and Vaucher 1991 for description of its type and only species), and *Emberizotaenia* Spasskaya, 1970 because of the presence of an unarmed rudimentary rostellum in their apical apparatus (see Georgiev and Genov 1993; Bona 1994). Moreover all these groups are parasitic in very specific groups of birds and, with the exception of *Cotylorhipis* and *Unciunia*, have never been recorded in South America. Our material is most similar to *Pseudochoanotaenia* Burt, 1938. However, *Pseudochoanotaenia* spp. are very small (up to 10 mm long) and our material is at least 2–3 times larger; they have a clear apical cavity in the glandular pouch which is lacking in our material; they have a short pyriform vagina versus a longer straight one in our specimens. Furthermore, *Pseudochoanotaenia* is presently restricted to Apodiformes and has never been reported from South America. In consequence, we consider the present material as belonging to a new genus.

### Janinellia peebeehi sp. n.

Taxon classificationAnimaliaCyclophyllideaDilepididae

http://zoobank.org/3D7F502C-EA02-4F1D-A45E-EEED2758FF59

Figures 1–5

#### Host:

*Elaeniachilensis* Hellmayr, 1927 (Passeriformes, Tyrannidae), Fio-fio, Chilean White-crested Elaenia.

#### Prevalence:

2/12 (16.7%).

#### Intensity:

1–2.

#### Site of infection:

Small intestine.

#### Locality:

HSFS, Comau Fjord, Los Lagos region, Chile, −42.39, −72.42. Altitude 10–30 m. (Type locality).

#### Date:

29.11.2008 and 2.12.2008.

#### Specimens studied:

3 specimens. Holotype: MHNG-PLAT-82292 (on slide). Paratypes: MHNG-PLAT-120515; MHNG-PLAT-82293 (2 specimens, on slides).

#### Hologenophore (genseq-2 COI):

MHNG-PLAT-82292 [CHIL-002/C2] and MHNG-PLAT-120515 [CHIL-038/C2]. Partial COI sequence, Genbank MH663460 and MH663461.

#### Etymology.

The species name phonetically reminds one of the acronyms of the NSF program that was sponsoring the project (Planet Biodiversity Inventory, PBI).

#### Description.

Body of small to medium size, up to 34 mm long (inferred from fragmented specimen). Strobila with almost parallel margins, gradually expanding in posterior direction: immature, mature, pregravid and gravid proglottides up to 250, 550, 700 and 1025 wide, respectively. Maximum width achieved at level of early gravid proglottides. Most complete specimens consisting of up to 157 proglottides. Proglottides weakly craspedote, wider than long except for very last gravid ones, which can be up to twice longer than wide. Two pairs of osmoregulatory canals. Ventral canals up to 50 in diameter, with posterior transverse anastomosis in each proglottis. Dorsal ones up to 11 in diameter.

**Figures 1–5.**
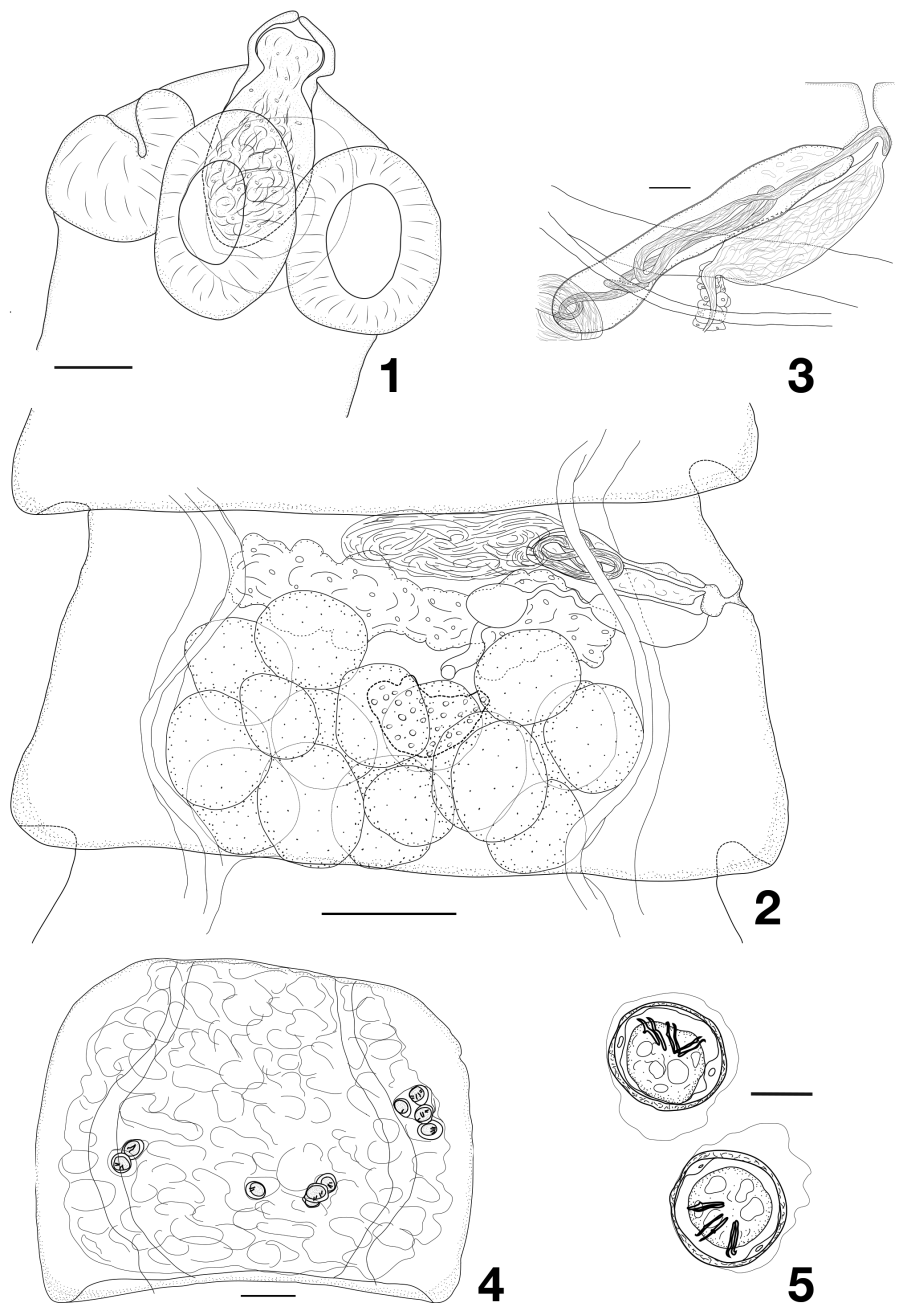
*Janinelliapeebeehi* gen. n., sp. n. **1** scolex **2** mature proglottis **3** cirrus sac **4** gravid proglottis **5** eggs. Scale bars: 50 µm (**1**), 100 µm (**2, 4**), 20 µm (**3, 5**).

Scolex (Fig. 1) not clearly separated from neck, 210–295 (258, *n* = 3) wide at middle of suckers; anterior part of scolex conically tapering; apex may form pipette-like protrusion. Suckers weakly muscular, oval 110–137 × 82–105 (120 × 96, *n* = 12). Apical apparatus consisting of oval rostellar pouch, 127–142 × 65–75, thin-walled, densely filled with glandular tissue, reaching to level of middle to posterior half of suckers. No rostellum. Neck straight, 225–325 wide. Genital ducts passing between osmoregulatory canals. Genital pores at border of first third of lateral proglottis margin, often forming genital papilla, irregularly alternating. Genital atrium simple, inconspicuous, thin-walled, tubular, with infundibular orifice.

Testes 13–17 (14.8, *n* = 25) in number, disposed in two dense layers, forming a continuous field filling most of posterior two thirds of median field of proglottides; posterior, lateral and dorsal to vitellarium, overlapping posterior parts of ovary; in younger proglottides, testes may occupy most of dorsal parenchyma (Fig. 2); testes 75–100 (89, *n* = 15) in diameter. External vas deferens very developed, forming multiple coils at extremity of cirrus-sac, filling the antero-poral and central part of median field. Cirrus-sac 130–182 × 25–38 (157 × 32, *n* = 30), oblique, straight, thin-walled, reaching or crossing poral osmoregulatory canals. Internal vas deferens coiled, making several loops in proximal half of cirrus-sac (Fig. 3). Evaginated cirrus short (up to 44 long), almost cylindrical, thin, 8–9 in diameter at its basal part, slightly tapering distally; unarmed.

Vitellarium large, up to 130 wide, central, lobate, oval or bean-shaped. Ovary transversely-elongate, massive and multilobulate, with two poorly marked and slightly asymmetrical wings, occupying entire width of median field and up to 30–40% of proglottis length. Mehlis’ gland not distinct as glandular structure. Ootype central, just anterior to vitellarium. Seminal receptacle oval 45–58 × 35–45 when empty, up to 150 × 87 when full. Vagina posterior to cirrus-sac, straight, distally thin-walled, proximally bordered with a row of large, dense cells; distal part often dilated, sometimes filled with spermatozoa (Figs 2, 3).

Uterus initially as loose reticulated network, then becoming denser as eggs develop, overlapping osmoregulatory canals and filling most of proglottis, including part of lateral fields (Fig. 4). Embryophore thick, 37–42 × 30–38 (39.5 × 34.5, *n* = 25). Oncosphere 27–30 × 22–27 (28.5 × 24, *n* = 25). Embryonic hooks sub-equal: central pair 13–15 (14, *n* = 10) long, lateral pairs 13–15 (13.5, *n* = 12) long (Fig. 5).

#### Remarks.

The new species is the type species of the newly erected monotypic genus *Janinellia* gen. n.

### Huinaylepis gen. n.

Taxon classificationAnimaliaCyclophyllideaDilepididae

http://zoobank.org/01B1BC94-EB49-4A9F-A145-A1A9B41F4D28

#### Type species.

*Huinaylepiselegans* sp. n. by original designation.

#### Diagnosis.

Dilepididae, Dilepidinae. Small strobila. Rostellum armed with two rows of hooks with peculiar and irregular 2-1 alternation. Rostellar pouch glandular. Suckers armed on anterior half, with largest antero-central hooks and progressively shorter ones laterally. Genital pores irregularly alternating. Genital ducts dorsal to osmoregulatory canals. Cirrus sac reaching osmoregulatory canals. Cirrus armed with strong spines. Testes in one field extending bilaterally and often also anteriorly to form circle reaching anterior proglottis margin. Ovary small. Uterus labyrinthic. Parasite of South American passerines (Furnariidae).

#### Etymology.

The genus name (feminine) derives from the name of the locality and the Latin suffix –lepis (scales).

#### Remarks.

Dilepidids with armed suckers, especially armed with true hooks and not merely spines, are very uncommon. The present material can only be compared with *Cotylorhipis* Blanchard, 1909, which is also found in South American furnariid birds. Although the general aspect of *C.furnarii* (Del Pont, 1906) and our specimens is similar because of the obvious sucker armament and despite an incomplete description of the former taxon [based on Del Pont (1906) as reported and completed in Blanchard (1909)], a number of characters easily separate them. *Cotylorhipis* specimens do not have any rostellum, their sucker armament is complete (all around the suckers circumference), and the testes never reach the anterior proglottis margin whereas our material shows an obvious armed rostellum in a large and distinctive rostellar pouch; sucker armament is restricted to the anterior half of suckers and testes are often far found anterior. Furthermore, the terminal gravid proglottides contain thousands of eggs and their length reaches up to 4–5 times their width in *Cotylorhipis*, while there are only a few hundreds of eggs and they are twice as long as wide in our specimens. These characters are sufficient to separate our material from *Cotylorhipis* and we propose to place it in the new genus *Huinaylepis*. It is, however, very likely that *Cotylorhipis* and *Huinaylepis* are closely related given the general appearance of their genital anatomy, presence of armed suckers, and shared host and geographical distribution.

### Huinaylepis elegans sp. n.

Taxon classificationAnimaliaCyclophyllideaDilepididae

http://zoobank.org/61C52012-B371-4B3A-B2D9-A2CD4E40678B

Figures 6–10 , 11

#### Host:

*Aphrasturaspinicauda* (Gmelin, 1789) (Passeriformes, Furnariidae), Rayadito, Thorn-tailed Rayadito.

#### Prevalence:

4/5 (80%).

#### Intensity:

4 to about 10 specimens.

#### Site of infection:

Small intestine.

#### Locality:

HSFS, Comau Fjord, Los Lagos region, Chile, −42.39, −72.42. Altitude 10–30 m. (Type locality).

#### Dates:

29.11–10.12.2008.

#### Specimens studied:

Holotype: MHNG-PLAT-82294 (on slide). Paratypes: MHNG-PLAT-82295; MHNG-PLAT-120512 to MHNG-PLAT-120514 (about 30 specimens, on slides). Additional non-type material: Locality: “Valdivia Forest Reserve, First refuge” (according to Franco Bona’s field books, deposited in MHNG). MHNG-PLAT-87937 16 slides from *Pygarrhichasalbogularis* (King, 1831) (Furnariidae), 19.01.1985, field number 657/5; MHNG-PLAT-87938, 5 slides from *Pygarrhichasalbogularis*, 20.01.1985. Field number 667/10; MHNG-PLAT-87939 14 slides from *Aphrasturaspinicauda*, 20.01.1985. Field number 672/12.

#### Hologenophore (genseq-2 COI):

MHNG-PLAT-120512 [CHIL-013/C2] and MHNG-PLAT-82295 [CHIL-071/C2 and C/4]. Partial COI sequence, Genbank MH663462 to MH663464.

#### Etymology.

The species name refers to the elegant (Latin: *elegans*) aspect of the worm’s scolex.

#### Description.

Body of small size, up to 18 mm long and with maximum width 600 at level of gravid or late mature proglottides. Up to 36 acraspedote proglottides (observed; maximum number of proglottides inferred from various fragments is about 44). Proglottides initially wider than long, progressively becoming as long as wide at level of male proglottides, then up to about twice as long as wide (terminal gravid proglottides). Development of strobila with marked steps instead of being progressive (similar to serial maturation sensu Spasskii 1951), i.e. with maturation of cirrus sac, testes or eggs sudden from one proglottis to next. Two pairs of osmoregulatory canals. Ventral canals with posterior transverse anastomoses in each proglottis.

**Figures 6–10.**
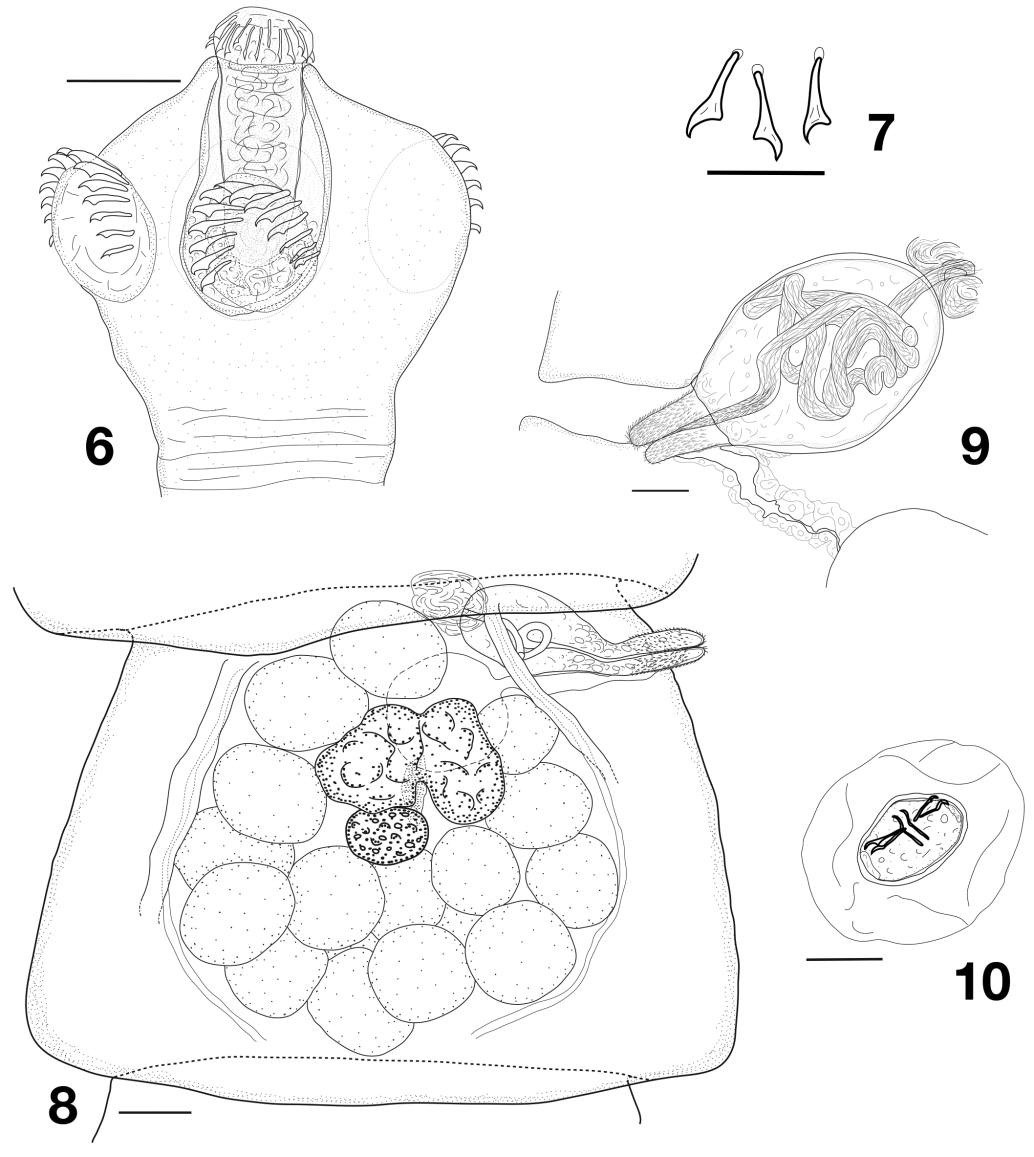
*Huinaylepiselegans* gen. n., sp. n. **6** scolex **7** rostella hooks **8** mature proglottis **9** cirrus sac **10** eggs. Scale bars: 40 µm (**6**), 20 µm (**7, 9, 10**), 100 µm (**8**).

**Figure 11.**
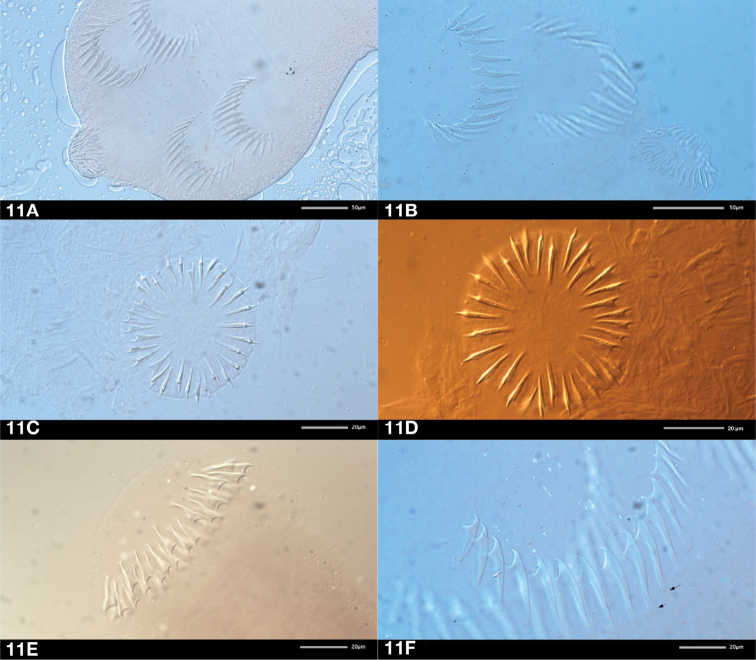
*Huinaylepiselegans* gen. n., sp. n. **A–B** rostellar armament **C–D** rostellar hooks, showing irregular alternations, apical views **E** lateral view of rostellar hooks **F** sucker hooks with epiphyses (arrows).

Scolex (Fig. 6) rounded. Rostellum musculo-glandular, mushroom-shaped, elongate, 67–112 × 40–55 (96 × 48, *n* = 18), with maximum diameter at level of crown of hooks. Rostellar pouch oval, 105–142 × 65–92 (126 × 78, *n* = 17), densely filled with glandular masses, reaching but usually not exceeding posterior margin of suckers. Rostellar hooks 29–35 in number, 16–18 long, in two rows, with particular 1-2 arrangement (1 hook on anterior row alternates with two on posterior row) but with recurring irregularities (i.e. 1-1-1, 2-2-2, 1-3-1) or sometimes intermediate positions (Fig. 11C–E). Hooks with long handle with small epiphyses, short blade and massive guard (Figs 7, 11E, F). Suckers round to slightly oval, with maximum diameter 60–90 (76, *n* = 79), bearing highly visible and very typical anterior semicircle of 12–17 (14.3, *n* = 109) hooks of similar shape as those of rostellum but with proportionally longer handle (Fig. 11A, B); epiphyses of sucker-hooks not always well marked but maybe conspicuous on handle (Fig. 11F); hooks of suckers up to 40 long, larger centrally and becoming gradually shorter (20–25) laterally (Fig. 11A B). Neck very short, formation of proglottides appears immediately behind scolex. Genital ducts dorsal to osmoregulatory canals. Genital pores alternating irregularly in short series, at border of first fifth of lateral proglottis margin.

Testes 16–22, rarely only 14 or up to 24 (19, *n* = 84) in number, disposed in entire median field reaching anterior proglottis margin and forming U-shaped field, with converging branches, often forming circle with one or two anterior testes linking the two lateral fields (Fig. 8), essentially in one layer, although some posterior testes may be superimposed; testes reaching up to 85 in diameter in late mature proglottides. Vas deferens coiled just at antiporal end of cirrus sac, often poorly visible but occasionally well-developed (up to 12 in diameter) and filling antero-central space. Cirrus-sac (Fig. 9) 90–137 × 50–77 (119 × 62, *n* = 55), ovoid, thin walled, anterior, with proximal extremity often touching anterior limit of proglottis, usually crossing poral osmoregulatory canals. Cirrus massive, armed with clearly visible spines (about 2 long) except on apex, 22–25 in diameter and reaching up to 145 long when evaginated. Internal vas deferens forming several coils.

Ovary central, small, compact, bilobed, anterior and dorsal to vitellarium. Vitellarium massive, central, compact, oval. Vagina posterior or ventral to cirrus pouch, straight, often dilated. Conductive part surrounded by thick but not dense cellular layer. Seminal receptacle oval, very large, commonly over 170 × 120, up to 217 × 160.

Young uterus saccular, becoming labyrinthic, filling space between osmoregulatory canals, containing a limited number (less than 200) of large eggs situated in 3 or 4 layers. Oncospheres ovoid, 29–37 × 19–25 (33 × 21.5, *n* = 21). Embryonic hooks 11–13 long (Fig. 10).

#### Remarks.

The new species is the type species of the newly erected monotypic genus *Huinaylepis* gen. n.

In 2005, the late Prof. Franco V. Bona (Torino) left his tapeworm collection to the MHNG. Among this rich material we found 35 slides of worms collected in *Aphrasturaspinicaudata* and *Pygarrhichasalbogularis*. All material was collected in the Valdivia Forest (about 250 km N of our study area) on 19–20 January 1985. On his accompanying notes, Bona noticed the interest of this material and indicated that it belonged to a new genus on some slides. Most of Bona’s specimens are juveniles, a few are mature and only a couple of proglottides are pregravid. We have examined all these slides and this materiel fully corresponds to *Huinaylepiselegans* sp. n. as described above. The only minor variations we noticed were two specimens with a slightly higher number of hooks than in the type series (36 and 38). These observations imply that *H.elegans* is probably more widespread in southern Chile than the few occurrences reported here may suggest. Over 30 species of Furnariidae are known in the country and, so far, have never been investigated for their parasites.

*Cotylorhipis* was a monotypic genus until Jadhav and Shinde (1981) described *C.sureshi* in domestic fowl from Aurangabad in India. Both the description and illustrations of this species are extremely succinct and of substandard quality. It is not even clear whether hooks reported by Jadhav and Shinde (1981) as “fanlike outgrowth”, and illustrated by asterisks, are present on suckers or not! In addition, and according to B. Jadhav (personal communication to M. Oros), there is no type material for *C.sureshi*. The disjoint geographic distribution, unrelated host, as well as the few morphological details given by Jadhav and Shinde (1981) make it highly dubious that their material belongs to *Cotylorhipis*. There is no possibility of checking the validity or taxonomic position of the material described as *Cotylorhipissureshi*, which must consequently be considered a species inquirenda.

### Kintneria

Taxon classificationAnimaliaCyclophyllideaDilepididae

(?) sp.

Figures 12–14

#### Host:

*Xolmispyrope* (Gmelin, 1789) (Passeriformes, Tyrannidae), Diucon, Fire-eyed Diucon.

#### Prevalence:

1/1.

#### Intensity:

3 specimens.

#### Site of infection:

Small intestine.

#### Locality:

HSFS, Comau Fjord, Los Lagos region, Chile, −42.39, −72.42. Altitude 10–30 m.

#### Date:

1.12.2008.

#### Specimens studied:

MHNG-PLAT-87930.

#### Hologenophore (genseq-2 COI):

MHNG-PLAT-87930 [CHIL-028/C2]. Partial COI sequence, Genbank MH663465.

#### Partial description

**Figures 12–15.**
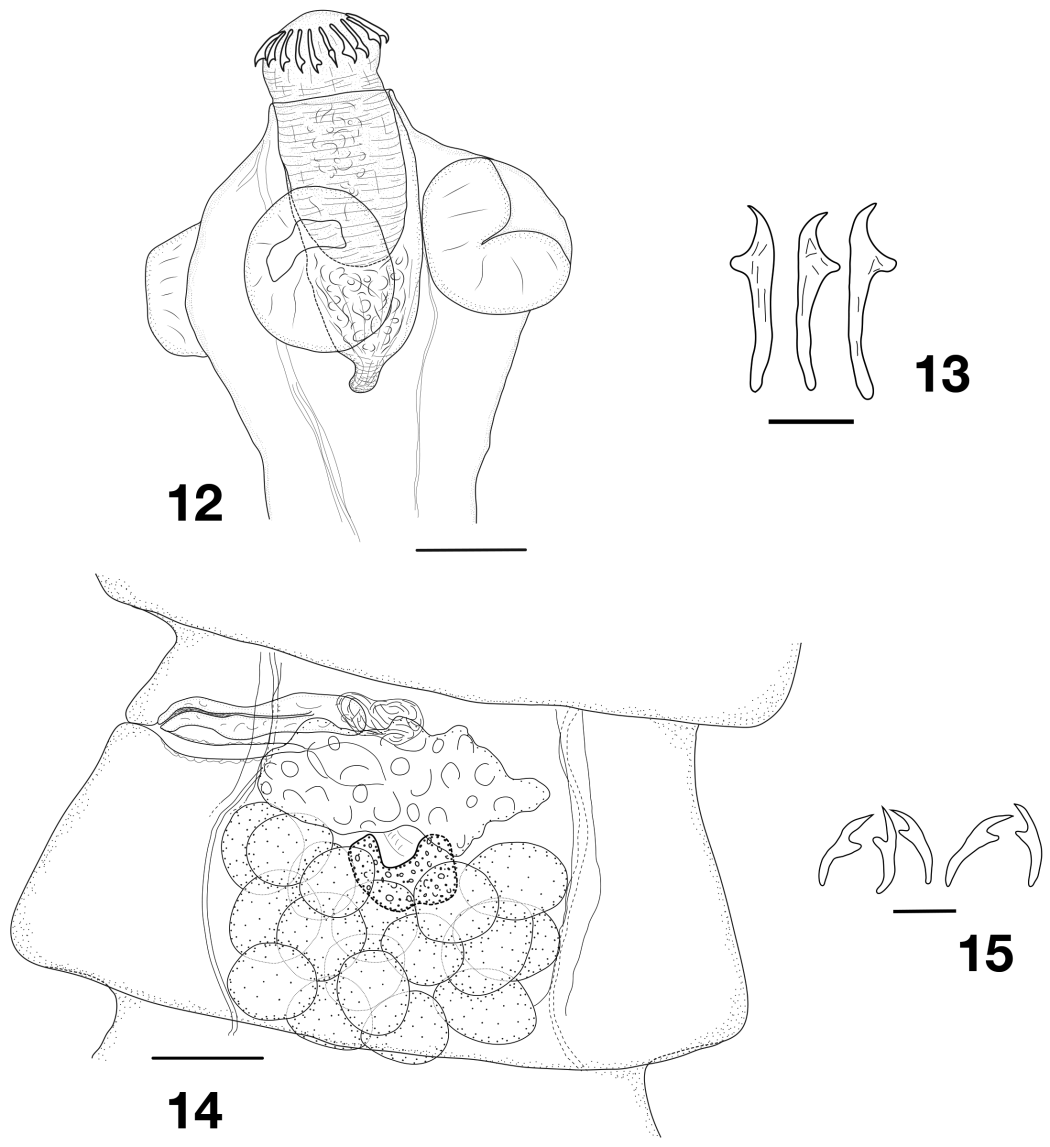
*Kintneria* (?) sp. **12** scolex **13** rostellar hooks **14** mature proglottis. Dilepididae gen. sp. 2 **15** rostellar hooks. Scale bars: 100 µm (**12, 14**), 20 µm (**13, 15**).

(based on only a few available mature proglottides): Body of small size, largest specimen 12.8 mm long and maximum width 675 mm wide. Up to 86 proglottides observed (up to mature stage, no complete specimens available), wider than long, craspedote. Scolex 275–330 (*n* = 2) in diameter, bearing four elongated and rather weak unarmed suckers155–175 (164, *n* = 7) in diameter. Rostellar pouch 280–285 × 123–148 (*n* = 2) with dense posterior glandular zone. Rostellum large, 243–265 × 95–118 (*n* = 2) present, strongly muscular but with distinct central glandular zone (Fig. 12). Rostellar hooks 17–18 (*n* = 2) in number, in one row, 41–47 long (44.5, *n* = 17), with long handle (Fig. 13). Neck well marked 162–250 (206, *n* = 3) wide. Formation of proglottides distinct at 242–312 (277, *n* = 3) from posterior margin of suckers. Genital pores situated in first third of length of lateral proglottis margin, alternating irregularly in small series (i.e. 3, 2, 1, 3), up to 6 consecutive pores on same side observed. Ventral osmoregulatory canals connected posteriorly in each proglottis by transverse anastomosis. Genital ducts passing between osmoregulatory canals.

Testes 16–21 (18, *n* = 10) in number, situated in single posterior field, in 2–3 layers, not extending beyond osmoregulatory canals. External vas deferens coiled, forming compact aggregation. Cirrus sac thin-walled 139–168 × 30–36 (149 × 33, *n* = 7) extending past the osmoregulatory canals. Cirrus armed with very fine spines.

Vitellarium immediately anterior to testes field, central, slightly V-shaped. Ovary lobulated and elongate transversely, anterior (poorly visible in our material). Vagina in same plane as, and posterior to, cirrus sac; wide, straight, opening in simple genital atrium about 15 deep (Fig. 14). No gravid proglottides and no early uterine development visible.

#### Remarks.

This material is likely to represent a new species. However a complete description is not possible without observations of the uterine development. Its generic position remains uncertain. According to Bona’s (1994) keys, it could belong to one of two very similar genera: *Monosertum* Bona, 1994 or *Kintneria* Spaskii, 1968, both parasitic in passerine birds. These genera differ from one another essentially by the structure of their uterus, with or without capsules, a character, which we cannot determine in the present material due to the lack of gravid proglottides. A few other diagnostic characters are given by Bona (1994). Among them, the small size of the body of our specimens rather resembles *Monosertum*; however, this character can often vary among species in many dilepidid genera. On the other hand, it corresponds to the diagnosis of *Kintneria* because of its larger rostellar hooks, the ovary that doesn’t reach the anterior proglottis margin and its cirrus armament consisting of short spines. For these reasons, we consider that it most likely belongs to the genus *Kintneria*. This genus was erected by Spasskii (1968) for a parasite of introduced European sparrows *Passerdomesticus* (L.) in North America. The type species of this genus was identified as *Choanotaeniapasserina* (Fuhrmann, 1908) by Kintner (1938). Spasskii (1968) considered it a distinct species, differing from the Palaearctic *Monopylidiumpasserinum* (Fuhrmann, 1908); he also placed it in the newly erected subgenusKintneria Spasskii, 1968 within the genus *Monopylidium* as M. (Kintneria) capsulata Spasskii, 1968. Bona (1994) eventually elevated *Kintneria* to the generic rank and its validity was accepted by Mariaux et al. (2017). *Kintneria* is known from the Nearctic and members of *Xolmis* are restricted to the Neotropics; however, other tyrant-flycatchers are known to migrate between North and South America, suggesting that genera of avian cestodes may have rather Pan-American than restricted distributions. This is the first cestode ever reported from the genus *Xolmis*.

To our knowledge the genus is monotypic and *K.capsulata* can easily be separated from our material by its shorter rostellar hooks and longer cirrus sac. Thus, should the observation of gravid segments confirm the placement of the present material into *Kintneria*, it would belong to a new species.

The two species belonging to the other similar genus, *Monosertumparinum* (Dujardin, 1845) and *M.mariae* (Mettrick, 1958), are known from European passerine birds only; furthermore, they are characterized by an osmoregulatory system forming a complicated reticular formation in the scolex and the neck (Komisarovas and Georgiev 2007). Such complicated network of osmoregulatory canals has not been observed in the present material.

## Dilepididae from Rhinocryptidae

A few specimens were retrieved from 2 species of terrestrial Rhinocryptidae with a limited distribution. Unfortunately, our material does not allow for complete descriptions of these worms. The limited available characters are briefly reported below.

**Dilepididae. gen. sp. 1**: Three fragments of 2 or 3 incomplete specimens, no gravid proglottides. Max length 2.6 mm for 20 proglottides weakly craspedote and wider than long, max width 560. A single scolex 312 wide. Powerful suckers 195–205 in diameter. Rostellar pouch indistinct, about 135 long. Rostellum muscular 107 × 40 with hooks mostly lost. Remaining hook fragments suggest 2 rows and a length of about 12. Neck short. Very rapid development of strobila with testes appearing in 7^th^ proglottides and mature proglottides in 12^th^ to 14^th^. Genital pores regularly alternating and genital ducts passing between osmoregulatory canals. 10–14 testes (12, *n* = 10) in one posterior field and 1 to 2 layers, not extending past osmoregulatory canals. Cirrus pouch 155–168 × 28–38, crossing excretory canals, slightly oblique. Cirrus armed with small spines. Ovary transverse anterior, bi-winged. Vitellarium posterior central and massive up to 58 × 96. Vagina posterior to cirrus pouch, finely armed.

MHNG-PLAT-120516. Host: *Scelorchilusrubecula* (Kitlitz, 1831) (Passeriformes, Rhinocryptidae), Chucao, Chucao Tapaculo. Small intestine. Prevalence: 1/6 (17%). Locality: HSFS, Comau Fjord, Los Lagos region, Chile, −42.39, −72.42, 50 m, 7.12.2008.

**Dilepididae gen. sp. 2** (Fig. 15): A single immature specimen with scolex. Scolex 20 in diameter, suckers circular 100–115 in diameter. Rostellum pouch 230 × 80, tapered and elongated posteriorly, with glandular mass at suckers level. Rostellum 152 × 70 strongly muscular. 20 subequal hooks 27–30 in length on 2 rows. Blade long, strong guard and handle slightly curved (Fig. 15). Neck short. About 15 immature proglottides with no anatomy visible.

MHNG-PLAT-120517. Same host and collection data as previous material.

**Dilepididae gen. sp. 3**: A single specimen in 2 parts, no scolex. Length 1.55 mm and 675 maximum width at gravid proglottides level. 75 proglottides, craspedote, mostly wider than long, becoming slightly longer than wide when gravid. Genital pores irregularly alternating in short series. Genital ducts in between osmoregulatory canals. Cirrus pouch 155–205 × 23–31, oblique, crossing osmoregulatory canals opening at anterior third of proglottides margin. Cirrus armed with small spines. Testes 12–16, in 2 layers, in a compact posterior field, in between osmoregulatory canals. Ovary bi-winged, anterior, difficult so see. Vitelline gland central, massive, up to 130 in diameter. Vagina posterior to cirrus pouch, copulatory part lined with short hairs/spines. Seminal receptacle large, central, anterior to vitellarium. Uterus multilobate, progressively filling entire proglottides. Eggs with thick external envelope.

MHNG-PLAT-120518. Host: *Pteroptochostarnii* (King, 1831) (Passeriformes, Rhinocryptidae), Black-throated Huet-huet, Hued-hued del sur. Small intestine. Prevalence: 1/5 (20%). Locality: HSFS, Comau Fjord, Los Lagos region, Chile, −42.39, −72.42, 10 m, 10.12.2008.

### Remarks

It is the first record of tapeworms in this family of birds. Despite the limited material available, key characters confirm that at least 3 different species of cestodes are present. Thus an interesting diversification of dilepidids has occurred, at least locally, in these rather elusive hosts. It would obviously be very interesting to collect more of them in various areas of their range, which encompasses most of South America.

## Discussion

It is widely accepted that biodiversity is particularly important in tropical rainforests (Mittermeier et al. 2003). However, our observations in Chile serve as a reminder that other biodiversity hotspots might be of great interest for parasitologists. This is particularly the case of Mediterranean-climate regions in general, and those with unique ecological conditions like the Chilean coastal forests (i.e. Ormazabal 1993; Smith-Ramirez 2004). In the humid temperate Chilean forests we have explored, the local diversity of birds is relatively poor, and we could only sample a small number of hosts. Nevertheless, not only the global prevalence (at 36% vs about 20% on average for similar studies according to our experience) and diversity of tapeworms in these hosts was high but their originality was also exceptional. *Elaeniachilensis* for example was previously found to harbour another new and, until now, endemic cestode species, *Anonchotaeniaprolixa* Phillips, Georgiev, Waeschenbach & Mariaux, 2014 (Phillips et al. 2014). The presence of several new endemic lineages (that may even be more numerous once the study of the remaining material from this mission is achieved) is remarkable for the region, and should stimulate more research in similar non-tropical areas. Although more observations are desirable before generalizing these results, they already confirm the interest of the Chilean Valdivian Temperate Forest as a biodiversity hotspot as recognized by Myers et al. (2000). Although some of these forests are relatively well protected compared to those in other parts of the world, parts of them remain highly vulnerable to progressive fragmentation and other human driven influence (Wilson et al. 2005; Echeverria et al. 2008; Schmitt et al. 2009; Gillespie et al. 2012). This is also an opportunity to point out that active research programs on parasites should be part of all studies involving the collection of vertebrate specimens in such areas.

## Supplementary Material

XML Treatment for Janinellia

XML Treatment for Janinellia peebeehi

XML Treatment for Huinaylepis

XML Treatment for Huinaylepis elegans

XML Treatment for Kintneria
